# LSCA-RCNN: Large-Kernel Spatial Residual and Cascade Attention Network for Voxel-Based 3D Object Detection

**DOI:** 10.3390/s26134089

**Published:** 2026-06-27

**Authors:** Yuyang Liu, Zhanyuan Jiang, Min Mao, Kun Zhang, Yu Xu, Mingchen Zhu, Xianjun Wu

**Affiliations:** 1 College of Physics and Electronic Engineering, Xinyang Normal University, Xinyang 464000, China; jiangzhanyuan2002@163.com (Z.J.); zk@xynu.edu.cn (K.Z.); xuyu20084636@126.com (Y.X.); 2 School of Information and Communication Engineering, University of Electronic Science and Technology of China, Chengdu 611731, China; 3 Xinyang Gumai Optronics Co., Ltd., Xinyang 464000, China; 4 School of Geomatics Science and Technology, Nanjing Tech University, Nanjing 210009, China; zhu_mc@njtech.edu.cn; 5 Xinyang Central Semiconductor Technology Co., Ltd., Xinyang 464000, China

**Keywords:** lidar point cloud, spatial residual blocks (SRBs), spatial-wise convolutions, ConvNeXt-based 2D backbone, 3D object detection, cascade detection head

## Abstract

LiDAR-based 3D object detection remains challenging due to sparse and irregular point cloud distributions, which degrade detection accuracy for small and occluded objects. In view of this, this paper proposes a novel two-stage voxel-based 3D detector, namely LSCA-RCNN, to address these issues. First, spatial residual blocks (SRBs) and large-kernel spatial-wise convolutions are integrated into the 3D backbone to suppress feature degradation and to expand the receptive fields for stable multi-scale feature learning. Second, a ConvNeXt-based 2D backbone with spatial attention is constructed to enhance discriminative feature representation of small objects. Third, a cascaded detection head embedded with fine-grained grouped convolutions and cross-stage cross-attention is designed to achieve progressive bounding box refinement and to improve localization precision. Extensive evaluations on the KITTI dataset with the R40 metric show that the proposed method achieves consistent performance improvements over the baseline. In the moderate setting, LSCA-RCNN increases the 3D AP by 2.12%, 7.66%, and 5.43% for cars, pedestrians, and cyclists, respectively, while achieving gains of 1.62%, 5.05%, and 7.05% under the hard setting. These results validate the effectiveness and robustness of the proposed LSCA-RCNN for complex and challenging autonomous driving detection tasks.

## 1. Introduction

Recent advances in autonomous driving technologies have made LiDAR a core sensor for environmental perception. 3D object detection serves as a fundamental task in autonomous driving, robotic navigation, and intelligent transportation systems, enabling intelligent platforms to perceive and understand spatial scenes accurately [1]. As a high-precision active sensing modality, LiDAR reliably captures the geometric coordinates, reflection intensity, and structural characteristics of outdoor environments, providing robust perceptual cues for complex driving scenarios [2]. However, LiDAR point clouds are inherently sparse and irregular, with spatially uneven density and significant cross-object scale variations. These intrinsic properties pose major obstacles for accurate and robust 3D object detection and limit the practical deployment of detection algorithms in real-world complex scenarios.

The existing LiDAR-based 3D object detection methods fall into three dominant categories: point-based, voxel-based, and hybrid point-voxel approaches. Point-based models, including PointNet [3], PointNet++ [4], PointRCNN [5], and 3DSSD [6], process raw point clouds directly and preserve fine-grained geometric information. Nevertheless, these methods incur substantial computational overhead, which constrains their real-time performance on large-scale point cloud data. By contrast, voxel-based methods discretize unstructured point clouds into regular 3D voxel grids and adopt sparse 3D convolutions for efficient spatial feature extraction, with representative architectures, such as SECOND [7], VoxelNet [8], and Voxel R-CNN [9]. Hybrid point-voxel methods exemplified by PV-RCNN and PV-RCNN++ combine voxel-based feature encoding with point-level localization to balance detection accuracy and computational efficiency [10,11]. Even so, their multi-branch architecture introduces inherent feature redundancy and training optimization instability, restricting further performance gains and practical deployment.

Voxel R-CNN is a high-performance two-stage 3D detector that employs voxel features for both proposal generation and bounding box refinement. It avoids dense point sampling and complicated multi-source feature aggregation, yielding high inference efficiency. Owing to structured voxel representation and sparse 3D convolutions, Voxel R-CNN has been widely adopted as a benchmark in both academic research and industrial applications. Despite its merits, this method still suffers from noticeable performance degradation in complex traffic scenarios. First, the backbone network lacks effective residual feature propagation. As network depth increases, geometric details and edge features of small and distant objects gradually decay, resulting in feature degradation. This issue severely impairs the detection of partially occluded pedestrians and distant cyclists, leading to frequent missed detections and low classification confidence. Second, the bird’s-eye view (BEV) feature extraction module lacks adaptive feature calibration. Standard convolutions fail to enhance foreground responses or to suppress background noise adaptively, thereby reducing feature discriminability for small and overlapping objects. Third, the single-stage proposal refinement mechanism has inherent limitations. It cannot fully correct coarse localization errors produced by the region proposal network (RPN). The accumulated regression errors further deteriorate localization accuracy in densely distributed and heavily occluded scenes, increasing false-positive detection rates.

To address the above limitations, this work proposes a novel 3D detection framework named LSCA-RCNN. The proposed framework integrates a spatial convolution-enhanced backbone optimized by SRBs, a ConvNeXt-based 2D backbone, and an attention-guided cascaded refinement strategy to achieve accurate and robust 3D object detection [12].

The main contributions of this paper are summarized as follows:

1. We propose a multi-scale residual 3D backbone composed of SRBs and spatial wise convolution modules to alleviate feature degradation by strengthening cross-layer feature propagation and by enhancing multi-scale contextual modeling with enlarged receptive fields.
2. We design a ConvNeXt-based 2D backbone with large scale spatial perception and lightweight spatial attention to enlarge the effective receptive field, to improve small object representation, and to enhance feature learning stability.
3. We propose a cascade component aware detection head with progressive cascade refinement and cross-stage cross-attention to improve fine grained feature perception and to reduce localization errors in single stage RoI prediction.
4. Extensive experiments on the KITTI dataset demonstrate the effectiveness and robustness of the proposed method.

## 2. Related Work

### 2.1. Voxel-Based 3D Detection

Voxel-based detection methods transform irregular point clouds into regular voxel grids, enabling convolutional networks to efficiently learn spatial features. Due to their favorable trade-off between accuracy and efficiency, voxel-based frameworks have become mainstream solutions for LiDAR-based 3D perception in autonomous driving. VoxelNet first introduced the voxel feature encoding (VFE) pipeline for end-to-end 3D object detection [8]. However, its dense 3D convolutions incur heavy computation on empty voxels, limiting the inference speed. SECOND addressed this issue by adopting sparse 3D convolutions to skip invalid voxels, significantly improving efficiency [7]. PointPillars further simplified voxel representation into 2D pillar features, achieving real-time detection performance [13]. These methods mainly belong to single-stage detectors, which emphasize speed but often struggle with small and occluded objects.

Two-stage voxel detectors instead focus on localization accuracy. Voxel R-CNN proposed a fully voxel-based two-stage framework with voxel RoI pooling, achieving a strong balance between accuracy and efficiency [9]. Nevertheless, it still suffers from two limitations: the backbone lacks effective residual connections, causing feature degradation in deep layers, and small convolution kernels provide insufficient receptive fields for capturing long-range spatial dependencies. Although methods such as JPV-Net employ multi-scale feature fusion to improve representation [14], they do not fundamentally resolve deep feature attenuation or limited receptive fields.

To address these limitations, this work reconstructs the 3D backbone of Voxel R-CNN by integrating spatial residual blocks (SRBs) and large-kernel spatial convolutions, thereby enhancing cross-layer feature propagation and enlarging the effective receptive field simultaneously.

### 2.2. Optimization of 3D Feature Enhancement

Feature representation is crucial for 3D object detection performance. The existing feature enhancement methods are mainly based on residual learning and attention mechanisms. Residual learning improves gradient propagation and preserves low-level spatial details through skip connections [15]. However, most residual designs in LiDAR detection are tailored for hybrid point-voxel frameworks, such as PV-RCNN [10,11], while pure voxel-based two-stage detectors rarely incorporate dedicated residual structures. As a result, Voxel R-CNN suffers from the progressive loss of fine-grained features for pedestrians and cyclists in deep networks.

Attention mechanisms adaptively recalibrate features, suppress background noise, and emphasize foreground targets [16]. Representative works include SENet [17], TANet [18], Bi-Att3DDet [19], and LoGoNet [20], which extend the attention mechanisms to multi-scale and multimodal scenarios. Nevertheless, most existing approaches focus on channel attention or modality fusion, while the spatial attention for BEV feature maps remains insufficiently explored. Since the BEV features encode critical spatial localization information, the lack of spatial calibration limits the discrimination of small objects from background clutter.

To overcome these issues, we optimize feature representation from two aspects. First, SRBs and large-kernel spatial-wise convolutions are embedded into the 3D sparse backbone to alleviate feature degradation and to enlarge receptive fields [21,22]. Second, a ConvNeXt-based 2D BEV encoder with spatial attention is constructed to enhance small-object representation and to improve the robustness of the BEV feature learning [12].

### 2.3. Proposal Refinement and Cascade Optimization

Accurate localization in two-stage detectors depends heavily on proposal quality and iterative refinement. Conventional frameworks use only a single-stage regression head to refine the RPN proposals, making it difficult to fully correct localization errors, especially in dense and occluded scenes [23].

Cascade refinement progressively optimizes proposals through multiple regression stages. 3D Cascade R-CNN improves localization by increasing the IoU thresholds across stages [24], while CasA introduces attention-based cross-stage feature aggregation for better refinement [25]. However, the existing cascade methods are mostly designed for hybrid point-voxel architectures and are not well suited to the pure voxel-based structure of Voxel R-CNN. Moreover, they lack fine-grained modeling for small objects, limiting their effectiveness for pedestrians and cyclists.

To address these shortcomings, we design a voxel-oriented cascaded detection head tailored to Voxel R-CNN. The proposed module combines multi-stage progressive regression with cross-stage cross-attention to reduce any accumulated localization errors. In addition, fine-grained grouped convolutions and component-aware mechanisms are introduced to enhance local structural perception for small targets, leading to more accurate localization in complex driving scenarios.

## 3. Methods

**Figure 1:**
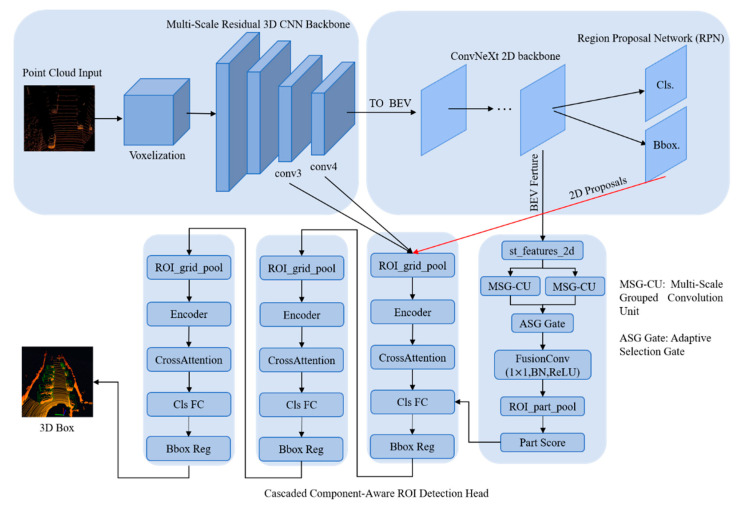
Overall architecture of the LSCA-RCNN. The framework consists of a multi-scale residual 3D CNN backbone with SRBs and large-kernel spatial convolutions for robust feature propagation, a ConvNeXt 2D backbone with spatial attention for enhanced BEV encoding, and a cascaded component-aware RoI head with three-stage progressive refinement and an auxiliary FGCE branch with MSG-CU and ASG for part-aware scoring.

To address the limitations described above, this paper proposes a novel 3D object detection framework, named LSCA-RCNN. The proposed framework integrates a multi-scale residual 3D backbone, a large-scale spatial perception BEV encoder, and a cascade component-aware RoI detection head. These elaborately designed modules collaboratively alleviate the deep feature degradation problem, while substantially enhancing the multi-scale contextual representation capability and object localization accuracy. The overall architecture of the proposed LSCA-RCNN framework is illustrated in Figure 1, which consists of three core functional components.

### 3.1. Multi-Scale Residual 3D CNN Backbone Network

**Figure 2:**
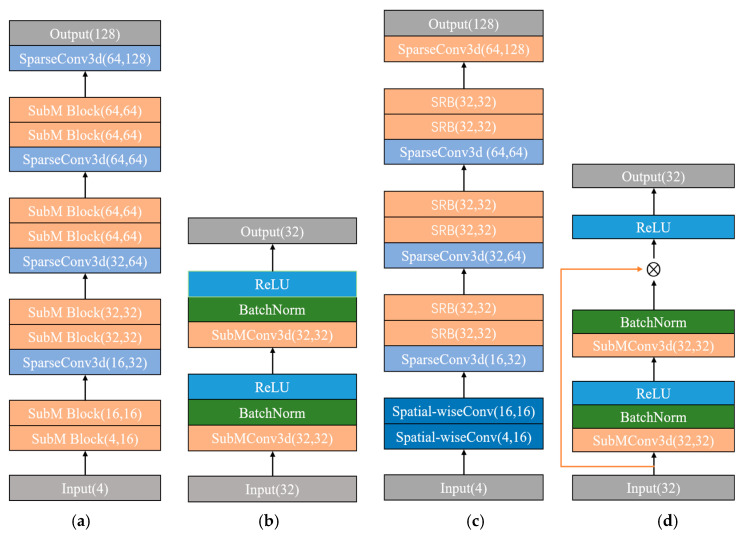
Comparison of backbone structures, where (**a**,**b**) illustrate the modules of Voxel R-CNN and (**c**,**d**) show the structures of the proposed method: (**a**) 3D backbone structure of Voxel R-CNN; (**b**) SubM block; (**c**) Multi-scale residual network structure; (**d**) Spatial Residual Block (SRB).

The baseline Voxel R-CNN adopts a 3D sparse convolutional backbone stacked with multi-level SubM blocks [9]. Each block contains two 3 × 3 × 3 SubM sparse convolution layers followed by batch normalization (BN) and ReLU activation. However, this sequential architecture suffers from several limitations. Sparse voxel features tend to degrade during deep feature propagation, resulting in weakened representations for small objects, such as pedestrians and cyclists. Moreover, small convolution kernels provide limited receptive fields and are insufficient for capturing long range spatial dependencies in point clouds. Although large convolution kernels can capture long range context, they significantly increase the computational cost and model complexity. To address these limitations, we design an improved 3D sparse convolutional backbone network, as shown in Figure 2. Dedicated enhancement modules are introduced for both shallow and deep layers. These modules enhance the multi-scale contextual modeling and stabilize the deep feature propagation.

#### 3.1.1. Spatial Residual Block

To alleviate feature degradation in the baseline backbone, the spatial residual block (SRB) is introduced to replace the original vanilla SubM blocks [21]. As illustrated in Figure 2d, the SRB employs two consecutive submanifold sparse convolution layers as the main feature extraction branch. Meanwhile, an identity residual connection is incorporated to fuse shallow geometric information with high-level semantic representations across the network layers. The aggregated features are activated by a ReLU function, facilitating stable gradient propagation during training. By strengthening cross-layer information flow, the SRB effectively preserves geometric details and mitigates feature degradation in deep sparse convolutional networks. As a result, it enhances the representation capability for small objects and improves the overall robustness of the detector.

#### 3.1.2. Spatial Dimensional Convolutional Modules

**Figure 3:**
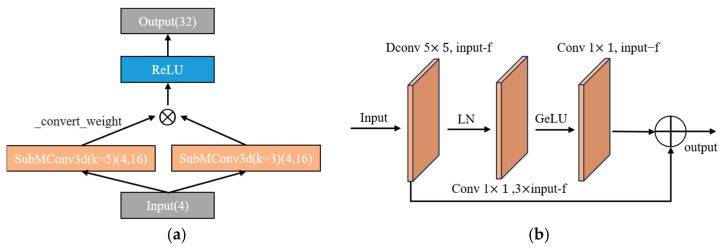
Illustration of key network modules: (**a**) Spatial-wise convolution module; (**b**) ConvNeXt module.

To strengthen the multi-scale spatial context modeling in the shallow feature encoding, a dual-branch parallel architecture is introduced in the shallow stages of the backbone network, as illustrated in Figure 3a. The two branches adopt 5 × 5 × 5 large-kernel convolutions and 3 × 3 × 3 SubMConv3d, respectively. A weight transformation mechanism is further introduced to enable parameter sharing across the multi-scale convolution kernels and to facilitate cross-branch feature fusion. The spatial-wise convolution module leverages large-kernel operations to expand the global receptive field and to capture long-range spatial dependencies within the sparse point cloud data [21,22]. Meanwhile, the embedded parameter sharing strategy eliminates redundant network parameters, reduces the optimization difficulty of large-kernel convolutions, and mitigates the risk of overfitting.

### 3.2. ConvNeXt 2D Backbone Network

The baseline model utilizes stacked 3 × 3 standard convolutions for the BEV feature encoding, which inherently suffers from constrained receptive fields, redundant computational overhead, unstable mini-batch training, and insufficient modeling capability for small-scale contextual features. To overcome these drawbacks, this paper proposes a ConvNeXt-based 2D backbone network, as depicted in Figure 3b. Derived from the classical ResNet architecture, ConvNeXt adopts 7 × 7 depthwise separable convolutions for hierarchical feature extraction [12,26,27]. Different from conventional standard convolutions, this design efficiently expands the network receptive field with limited computational and parameter costs, enabling superior capture of fine-grained features and contextual information for small objects.

Equipped with LayerNorm normalization, GELU activation functions, and learnable gamma scaling parameters, the proposed 2D backbone network mitigates gradient vanishing issues and stabilizes mini-batch training. Furthermore, a spatial attention module is embedded at the end of each network stage [28]. This module generates 2D spatial attention maps by aggregating the channel-wise average and maximum pooling features, which adaptively enhances foreground target responses and suppresses irrelevant background noise on the BEV feature plane. Additionally, a multi-scale feature fusion strategy is integrated into the framework. Hierarchical down-sampling operations are adopted for cross-stage feature compression and semantic encoding, while deconvolution-based up-sampling is employed to reconstruct the multi-scale BEV feature representations. The finally aggregated discriminative features provide reliable feature priors for subsequent RoI refinement, object classification, and bounding box regression tasks.

### 3.3. Cascaded Component Aware RoI Detection Head

**Figure 4:**
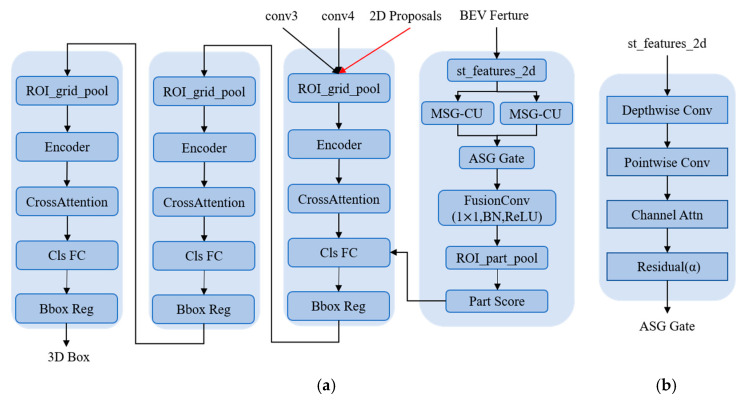
Structure of the cascaded component aware ROI detection head and multi-scale grouped convolution unit: (**a**) Cascaded component aware RoI detection head, consisting of the cascaded detection head and the fine-grained grouped convolutional component aware module; (**b**) Multi-scale grouped convolution unit (MSG-CU).

The conventional single-stage RoI prediction paradigm adopted in baseline detectors often suffers from localization errors and insufficient bounding-box refinement, particularly for small and sparsely distributed objects. To address these limitations, a cascaded component-aware RoI detection head is proposed, as illustrated in Figure 4a. Inspired by cascaded attention networks, the proposed design extends the cascaded optimization strategy to 3D LiDAR-based object detection by constructing a multi-stage attention-guided refinement framework within the RoI head [29].

The cascaded structure progressively refines object proposals and facilitates more effective feature learning across stages. In addition, the component-aware feature extraction mechanism enhances the perception of fine-grained structural information, leading to more discriminative object representations. Through iterative optimization, the proposed detection head improves the localization quality and feature refinement, thereby achieving a more robust detection performance for challenging targets, such as pedestrians and cyclists in complex traffic environments.

#### 3.3.1. Fine-Grained Grouped Convolutional Component-Aware Module

To strengthen the structural feature modeling of small targets, this work embeds a fine-grained part-aware branch into the cascaded detection head. Different from conventional standard convolution-based detection branches, this paper proposes a fine-grained group convolution enhancement (FGCE) module built upon depthwise separable convolutions. This module is specifically tailored for small objects (e.g., pedestrians and cyclists) that suffer from limited BEV projection areas, sparse point cloud distributions, and insufficient structural feature representation. Since standard convolutions fail to capture the subtle structural correlations of such small targets effectively, a multi-scale grouped convolution unit (MSG-CU) is further designed to refine fine-grained feature learning, with its detailed structure depicted in Figure 4b.

Given the input feature $F\in R^{B\times C\times H\times W}$, MSG-CU first splits the input channel dimension into G=16 disjoint groups: $F=\left[F^{\left(1\right)};F^{\left(2\right)};\cdots ;F^{\left(G\right)}\right]$, where each group feature satisfies $F^{\left(g\right)}\in R^{B\times \frac{C}{G}\times H\times W}$. Each group is independently processed via depthwise separable convolution to extract group-specific spatial features, formulated as follows:

(1) $$F_{dw}^{\left(g\right)}=W_{dw}^{\left(g\right)}*F^{\left(g\right)},\;g=1,2,\ldots ,G$$

where $W_{dw}^{\left(g\right)}\in R^{k\times k\times \frac{C}{G}}$ denotes the learnable depthwise convolution kernel, and the kernel size k∈(3,5) for multi-scale feature modeling. Subsequently, pointwise convolution is adopted to achieve adaptive cross-group feature fusion and to integrate multi-group complementary information, which can be expressed as follows:

(2) $${\;F}_{pw}=W_{pw}\cdot Concat\left[F_{dw}^{\left(1\right)},\ldots ,F_{dw}^{\left(G\right)}\right],W_{pw}\in R^{C_{out}\times C}$$

To enhance the channel-wise feature sensitivity and to suppress redundant channel responses, a channel attention mechanism is introduced to recalibrate the fused features, as follows:

(3) $$\omega_{c}=\sigma (W_{2}\cdot ReLU(W_{1}\cdot \frac{1}{HW}\sum_{i=1}^{H}\sum_{j=1}^{W}F_{pw}(i,j)))$$

where ${W_{1},W}_{2}\in R^{C_{out}\times \frac{C_{out}}{4}}$ denotes the linear projection layers. The recalibrated feature is obtained as $F_{ca}=F_{pw}⊙\omega_{c}$. Furthermore, a learnable residual scaling factor $\alpha =tanh(\alpha_{l})$ is incorporated, which stabilizes network training at the initial stage and adaptively regulates the degree of feature enhancement during optimization.

To address the inherent scale discrepancy between pedestrians and cyclists in the BEV feature maps, the FGCE module adopts a dual-branch multi-scale design. The 3 × 3 convolution branch captures fine-grained local structural features for pedestrian perception, while the 5 × 5 convolution branch leverages a larger receptive field to model the global structural characteristics of cyclists. An adaptive spatial gating mechanism $G=\sigma (W_{g}*F_{in})$ is employed to fuse dual-branch features, which dynamically adjusts the contribution of each branch according to spatial position variations. Finally, the component-aware perceptual features are integrated with geometric classification outputs to generate the final prediction results, providing explicit structural prior knowledge to facilitate the high-precision detection of small and easily confused traffic objects.

#### 3.3.2. Cross-Stage Cross-Attention

To facilitate efficient global feature propagation and to eliminate redundant computation across cascaded optimization stages, a shared feature connection layer is embedded at the input of the cascaded detection head. On this basis, a cross-attention mechanism is integrated to enable sufficient feature interaction and complementary learning between adjacent stages [30]. Let ${\hat{F}}^{j}$ and $F^{j}$ denote the feature maps derived from the previous and current cascaded stages, respectively. The cross-attention calculation is formulated as follows:

(4) $${\;\hat{F}}_{i}^{j}=softmax\left(\frac{Q_{i}^{j}{\left(K_{i}^{j}\right)}^{T}}{\sqrt{c^{\prime }}}\right)V_{i}^{j}$$

where $Q^{j}$, $K^{j}$, and $V^{j}$ represent the query, key, and value embeddings linearly projected from stage-specific features, respectively. This mechanism enables the network to adaptively model long-range dependencies across different cascaded stages, thereby achieving hierarchical feature aggregation and significantly improving feature representation robustness. The refined multi-stage interactive features are ultimately fed into the classification and regression branches to produce accurate 3D object detection results. The overall pipeline of the proposed cross-stage cross-attention module is illustrated in Figure 4a.

### 3.4. Loss Function

Unlike the original Voxel R-CNN, which employs a single-stage RoI refinement head, the proposed framework adopts a three-stage cascade refinement strategy. Accordingly, the overall training objective is formulated as the sum of the RPN loss and the losses from all cascade stages: $L=L_{RPN}+\sum_{k=1}^{K}L_{roi}^{\left(k\right)}$, where $L_{RPN}$ is the RPN loss, $L_{roi}^{\left(k\right)}$ denotes the loss of the (k)-th cascade stage, and (K = 3) is the total number of cascade stages. All cascade stages are equally weighted during training. The detailed formulations are defined as follows:

(5) LRPN=1Np∑iLclsai,a^i+λ1IIoUi>u∑iLregδi,δ^i

(6)  Lroik=1Nb∑jLclsajk,a^jk+λ2IIoUjk>uk∑jLregδjk,δ^jk

For $L_{RPN}$, $N_{p}$ denotes the number of anchors; $a_{i}$ and $\delta_{i}$ represent the predicted classification scores and bounding box offsets, while a^i and δ^i denote the ground-truth labels and targets; I(·) is the indicator function with u=0.5. $L_{cls}(\cdot )$; and $L_{reg}(\cdot )$ denotes the focal loss and smooth $L_{1}$ loss, with a balancing weight $\lambda_{1}=1.0$. For the *K*-th stage $L_{roi}^{\left(k\right)}$, $N_{b}$ denotes the number of RoIs; $a_{j}^{\left(k\right)}$ and $\delta_{j}^{\left(k\right)}$ are the predicted outputs, while a^j(k) and δ^j(k) are the assigned labels and targets.

## 4. Experiment

### 4.1. Dataset and Evaluation Metrics

The KITTI dataset is one of the most authoritative and widely adopted benchmarks for LiDAR-based 3D object detection in autonomous driving scenarios, owing to its diverse traffic scenes, high-precision annotations, and reliable data quality [31]. This study strictly follows the official data partition protocol of Voxel R-CNN for a fair comparison, dividing the entire trainval set into 3712 training samples and 3769 validation samples. Consistent with the official KITTI evaluation criteria, all test samples are classified into three difficulty levels (easy, moderate, and hard) according to the degree of object occlusion and shooting distance. In this work, 3D average precision (AP) with 40 recall points (R40) is adopted as the core evaluation metric [32]. Standard IoU thresholds are employed for quantitative evaluation, including 0.7 for car detection and 0.5 for pedestrian and cyclist detection tasks.

### 4.2. Experiment Details

All experiments are conducted on a server equipped with two NVIDIA GeForce RTX 3090 GPUs (24 GB VRAM) under the Ubuntu 20.04 LTS operating system. The programming language used is Python 3.6.9. The software environment is strictly unified for reproducibility: PyTorch 1.8.0, CUDA 11.1, cuDNN 8.0.5. The adopted third-party libraries include sparse 3D convolution spconv 2.1.25 and NumPy 1.21.6 and Open3D 0.11.0 Scikit-learn 1.3.2.

We adhere to the standard preprocessing workflow of Voxel R-CNN for the coordinate transformation and value normalization of raw LiDAR point clouds. To remove invalid distant points, we constrain all point coordinates within the range x∈[0, 70.4], y∈[−40, 40], z∈[−3, 1]. The voxelization hyperparameters are predefined: the voxel dimension is (0.05 m,0.05 m,0.05 m), the maximum number of points contained in a single voxel is 5, and the maximum voxel quantity is set to 16,000 for the training set and 40,000 for the test set [9]. A series of conventional augmentation strategies for LiDAR 3D detection are utilized during training only, namely gt_sampling, random world flip, random world rotation, random world scaling, and random local pyramid augmentation. These augmentations are disabled for validation and inference.

The network is trained over 80 epochs with a per-GPU batch size of 4, using the AdamW optimizer for parameter updating. For a rigorous and fair experimental comparison, all configurations, including hyperparameters, weight initialization rules, and random seed policies, strictly conform to the Voxel R-CNN baseline, and no extra hyperparameter adjustment is implemented in our experiments.

### 4.3. Comparison with Other Algorithms

To fully evaluate the performance of the proposed LSCA-RCNN, we conduct extensive comparative experiments on the KITTI validation set. The proposed framework is compared against several state-of-the-art 3D object detection approaches, with the quantitative results listed in Table 1 and Table 2. Additional comparisons with representative multimodal detectors, including Bi-Att3DDet, LoGoNet, SQD, SFD, and VirConv-L, are presented in Table 3 [19,20,33,34,35].

As shown in Table 1, LSCA-RCNN achieves 3D AP scores of 86.64%, 67.33%, and 76.37% for cars, pedestrians, and cyclists, respectively, under the moderate difficulty setting. Compared with the baseline Voxel R-CNN, the proposed method improves the 3D AP by 2.12%, 7.66%, and 5.43% for the three categories, demonstrating consistent accuracy gains while maintaining balanced performance across different object classes. Notably, LSCA-RCNN outperforms several representative two-stage LiDAR-based detectors, including PV-RCNN++, CasA, and CIA-3D, in pedestrian and cyclist detection, indicating its effectiveness in handling small and sparsely distributed objects.

To further assess the robustness of the reported results without incurring the high computational cost of multiple independent training runs (approximately 20 GPU-hours per run), a bootstrap resampling analysis is conducted on the predictions of a single trained model. Specifically, 3769 test samples are randomly sampled with replacement to generate N = 100 bootstrap datasets. The KITTI AP metric is recomputed for each dataset, and the final performance is reported as the mean ± standard deviation over all bootstrap iterations. As shown in the last row of Table 1, the proposed method achieves low performance variance across the different categories and difficulty levels, demonstrating the statistical reliability and robustness of the reported results.

Table 2 further presents the 3D and BEV detection results for the car category. LSCA-RCNN achieves 3D AP scores of 93.27%, 86.64%, and 84.16% under the easy, moderate, and hard settings, respectively. Under the moderate setting, it surpasses Voxel R-CNN by 2.12% in 3D AP and 1.02% in the BEV AP, demonstrating its strong capability in spatial feature representation and object localization. Although some methods, such as SA-Voxel R-CNN, and TED, achieve slightly higher 3D AP under the hard setting, LSCA-RCNN still maintains a competitive 3D AP of 84.16% while achieving a BEV AP of 89.90%. Moreover, it delivers a consistently strong performance across all evaluation settings, indicating a favorable balance between localization accuracy and overall detection robustness.

By jointly analyzing the results in Table 1 and Table 2, we further investigated the effectiveness of LSCA-RCNN across different object categories and the factors influencing its detection performance. Compared with Voxel R-CNN, the proposed method achieves consistent improvements for cars, pedestrians, and cyclists. However, the magnitude of the improvement varies across the categories.

Cars usually contain dense point clouds and well-preserved geometric structures, allowing most existing detectors to localize them accurately. As a result, most of the existing algorithms could achieve a higher performance for cars. By contrast, pedestrians and cyclists are considerably more challenging due to their small physical size, sparse point cloud distributions, frequent occlusions, and limited BEV projection areas. These characteristics often lead to incomplete feature representations and a reduced detection accuracy.

The larger improvements observed for pedestrians and cyclists demonstrate the effectiveness of the proposed architecture. By combining multi-scale feature extraction, enhanced BEV feature encoding, and cascaded RoI refinement, LSCA-RCNN can capture more discriminative fine-grained features and better preserve the structural information for small objects. Consequently, the proposed method achieves superior robustness and generalization capability when detecting challenging road users in complex autonomous driving scenarios.

As shown in Table 3, LSCA-RCNN is compared with several state-of-the-art multimodal detection methods. Although these approaches exploit the complementary information from both LiDAR and images, LSCA-RCNN, as a purely voxel-based single-modal framework, still achieves a competitive performance on multiple evaluation metrics. For pedestrian detection under the moderate setting, LSCA-RCNN achieves a 3D AP of 67.33%, which is comparable to Bi-Att3DDet (67.57%) and surpasses LoGoNet (63.72%). For cyclist detection, LSCA-RCNN attains 76.37% for 3D AP under the moderate setting, outperforming LoGoNet (75.35%) and remaining competitive with Bi-Att3DDet.

These results demonstrate the effectiveness of the proposed feature representation and component-aware detection strategy for challenging small-object detection tasks. Although multimodal methods generally benefit from richer complementary information, LSCA-RCNN achieves a comparable performance while relying solely on LiDAR point clouds. This indicates that the proposed voxel feature enhancement and cascaded detection framework can effectively exploit geometric information from sparse point clouds, providing a favorable balance between detection accuracy, model simplicity, and computational efficiency.

**Figure 5:**
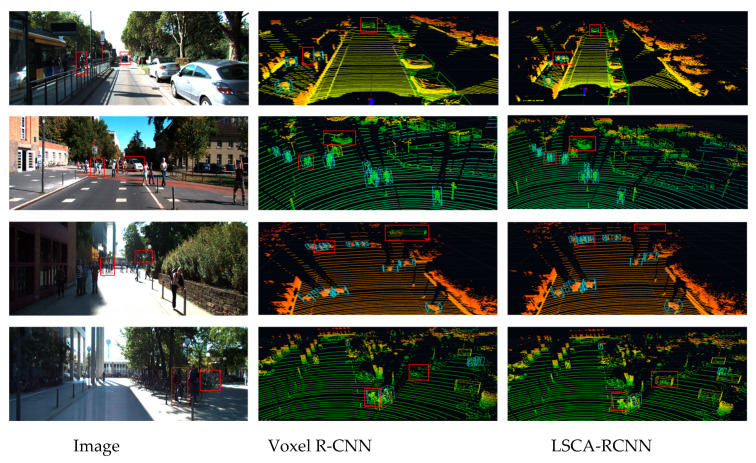
Visualization of detection results on the KITTI dataset. Blue boxes represent pedestrians, yellow boxes represent cyclists, and green boxes represent vehicles. The red box denotes the comparison region.

Figure 5 presents a qualitative visualization comparison between the proposed LSCA-RCNN and the baseline Voxel R-CNN, with the samples arranged as ground truth, Voxel R-CNN, and LSCA-RCNN from left to right. The visualization results intuitively demonstrate the superior performance of LSCA-RCNN in complex traffic scenarios, especially for distant and occluded small objects. In the first two rows of visualization cases, the baseline Voxel R-CNN fails to detect distant vehicles, mid-road pedestrians, and bus stop-occluded objects, whereas LSCA-RCNN successfully identifies these challenging targets. Meanwhile, LSCA-RCNN effectively reduces false-positive detections. In the last two rows, Voxel R-CNN misclassifies distant tree shadows and roadside vegetation as cars and cyclists, while the proposed method suppresses such invalid interference and achieves accurate detection.

Although a small number of false negatives still exist in the detection results, LSCA-RCNN achieves a comprehensive performance improvement over the baseline model. The qualitative results further verify that the proposed modules can significantly enhance the model’s perception ability for sparse, small, and occluded objects in complex scenes, fully demonstrating the effectiveness and superiority of the proposed LSCA-RCNN framework.

### 4.4. Ablation Experiment

***Effectiveness of the proposed 3D backbone:*** To evaluate the effectiveness of different 3D backbone designs, we compared the original Voxel R-CNN with two backbone variants on the KITTI validation set, and the AP40 results for pedestrian and cyclist detection are reported in Table 4. The backbone incorporating only the spatial–dimensional convolution module (SDCM) achieves the highest cyclist detection accuracy across all difficulty levels, whereas its pedestrian detection performance is inferior to that of the spatial residual block (SRB)-based backbone. The SRB effectively alleviates feature degradation during deep feature propagation, resulting in more stable feature learning and superior pedestrian detection, although its gains for cyclists are relatively limited. To exploit the complementary strengths of both modules, we integrate an SDCM and an SRB into a unified backbone. The resulting hybrid architecture achieves the best pedestrian detection performance across all difficulty levels and consistently improves cyclist detection. Although it does not attain the highest score in every category, it delivers the strongest overall performance. These results demonstrate that the proposed backbone effectively enlarges the receptive field of shallow layers while mitigating deep feature degradation, thereby enhancing feature representation and improving detection accuracy.

***Effectiveness of the proposed detection head:*** To evaluate the effectiveness of the proposed detection head, we compared the original Voxel R-CNN with different detection head configurations. As shown in Table 5, the cross-stage cross-attention (CSCA) mechanism improves the detection performance by progressively refining object proposals through multi-stage optimization. However, the CSCA primarily focuses on bounding box refinement and provides limited enhancement for the sparse and incomplete structural features of pedestrians and cyclists. To address this issue, the fine-grained grouped convolutional component-aware (FGCA) module is introduced to strengthen fine-grained feature perception during RoI refinement. The results show that the combination of the CSCA and FGCA achieves the best cyclist detection performance across all difficulty levels and attains the highest AP40 for pedestrians under the hard setting. These improvements demonstrate that the proposed detection head effectively enhances component-level feature representation and fine-grained feature learning, leading to more accurate localization and recognition of challenging objects.

***Effects of different components:*** To validate the effectiveness of the proposed modules, comprehensive ablation studies were conducted on the KITTI validation set, with the results reported in Table 6. All experiments are built upon Voxel R-CNN and evaluated using AP40. Compared with the baseline, the proposed MSR3DCNN (Mutli-scale Residual 3D CNN) backbone significantly improves detection accuracy. As shown in Group A, the moderate AP40 increases by 1.54%, 6.23%, and 2.94% for cars, pedestrians, and cyclists, respectively, demonstrating the effectiveness of enlarged receptive fields and enhanced feature propagation. To further enhance the BEV feature extraction, a ConvNeXt-based 2D backbone is introduced. As shown in Group E, combining MSR3DCNN with ConvNeXt further improves pedestrian detection performance. However, the gains for cars and cyclists remain limited, indicating that the enhanced BEV features alone cannot be fully exploited without effective RoI refinement. Therefore, the cascaded component-aware RoI detection head (CasA_FGCE) is incorporated to improve the proposal refinement and structural feature learning. As shown in Groups C and D, CasA_FGCE consistently outperforms the baseline. Finally, the full model achieves the best overall performance, demonstrating the complementary advantages and strong synergy of the three proposed modules.

***Computational complexity analysis:*** To assess the computational efficiency of the proposed modules, Table 7 reports the parameter count and inference latency of different model configurations. The baseline MSR3DCNN backbone requires only 6.03 M parameters and 47.20 ms inference time. Adding the ConvNeXt-based BEV backbone introduces a slight increase in the computational cost, while the CasA_FGCE detection head contributes a larger overhead due to cascaded RoI refinement and feature enhancement. Nevertheless, both modules yield notable accuracy improvements. The full model achieves the best overall performance with 11.51 M parameters and 70.20 ms latency, attaining mean AP40 scores across the easy, moderate, and hard settings of 88.02%, 67.56%, and 80.00% for cars, pedestrians, and cyclists, respectively. These results indicate that the proposed framework provides a favorable trade-off between detection accuracy and computational efficiency.

## 5. Conclusions

This work presents LSCA-RCNN, a novel multi-stage voxel-based 3D detection framework for autonomous driving. To mitigate feature degradation and insufficient multi-scale perception in traditional voxel backbones, we embed SRBs and large-kernel spatial convolutions into 3D sparse convolutions, retaining the geometric details and enlarging the receptive fields. Combined with a ConvNeXt 2D backbone equipped with depthwise separable convolutions and spatial attention, the model strengthens the BEV feature representation and improves the detection of small and hard objects. Moreover, the cascaded RoI detection head with grouped convolution and cross-stage cross-attention refines the bounding boxes progressively, reducing localization errors for sparse and occluded targets. Evaluated on the KITTI dataset, LSCA-RCNN outperforms Voxel R-CNN in various scenarios and object classes. The experimental results confirm the efficacy and strong generalization of our method, which shows great robustness for sparse point clouds, distant objects, and occlusions for practical outdoor 3D detection. Nevertheless, the proposed LSCA-RCNN still has certain limitations, including degraded detection performance in extreme weather scenarios and slightly insufficient real-time inference speed due to increased computational overhead. In future work, we will optimize the network lightweight structure and introduce adaptive feature adjustment mechanisms to improve the model’s real-time performance and environmental generalization ability for practical autonomous driving deployment.

## Figures and Tables

**Table 1:** Comparison of Different Algorithms on the Validation Set. The bold results indicate the best performance. Note: Results marked with (*) are reproduced by our team under identical training, validation, and test configurations to ensure fair comparison; unmarked results are directly cited from the original published papers.

Model	Stage	Car.3D (APR40)			Ped.3D (APR40)			Cyc.3D (APR40)		
		Easy	Mod.	Hard	Easy	Mod.	Hard	Easy	Mod.	Hard
SECOND * [7]	1-stage	88.61	78.62	77.22	56.55	52.98	47.73	80.58	67.15	63.10
PointPillars * [13]	1-stage	86.46	77.28	74.65	57.75	52.29	47.90	80.05	62.68	69.70
VFL3D [36]	1-stage	91.89	85.11	82.64	59.90	53.35	49.11	84.05	65.86	61.97
PointRCNN * [5]	2-stage	91.78	80.72	78.22	60.01	53.38	45.78	90.60	71.80	67.45
Part A2 * [37]	2-stage	89.36	80.23	78.88	65.70	61.32	55.40	85.60	69.24	65.63
PV-RCNN++ * [11]	2-stage	90.85	83.66	81.56	63.36	57.55	53.11	87.93	69.77	65.97
CIANet [38]	2-stage	89.68	84.89	79.32	67.95	62.54	56.91	87.12	73.78	70.69
RCAVoxel-RCNN [39]	2-stage	92.81	85.78	83.51	71.46	64.74	58.72	**92.92**	**76.61**	71.62
CasA * [29]	2-stage	90.01	86.30	82.83	73.89	66.55	59.89	92.61	73.04	68.44
Voxel R-CNN * [9]	2-stage	92.24	84.52	82.54	65.51	59.67	53.73	90.72	70.94	66.88
**Ours**	2-stage	**93.27**	**86.64**	**84.16**	**74.57**	**67.33**	**60.78**	91.71	76.37	**71.93**
Bootstrap mean ± standard deviation		93.30 ±0.18	86.39 ±0.75	83.34 ±1.08	74.91 ±1.58	67.05 ±1.44	60.80 ±1.30	92.22 ±1.66	76.48 ±2.16	72.21 ±2.35

**Table 2:** Comparison of Different Algorithms for the Car Category on the KITTI Validation Set in terms of 3D and BEV Performance. The bold results indicate the best performance. Note: Results marked with (*) are reproduced by our team under identical training, validation, and test configurations to ensure fair comparison; unmarked results are directly cited from the original published papers. The symbol “-” indicates that the corresponding data were not reported in the original publication.

Model	Reference	Modality	Car.3D (APR40)			Car.BEV (APR40)		
			Easy	Mod.	Hard	Easy	Mod.	Hard
PV-RCNN++ * [11]	IJCV 2023	LiDAR	90.85	83.66	81.56	92.90	89.64	88.79
CasA * [29]	IEEE TGRS 2022	LiDAR	90.01	86.30	82.83	93.93	90.22	87.74
TED [40]	AAAI 2023	LiDAR	92.59	85.81	**85.26**	95.05	91.43	91.22
SA-Voxel-RCNN [41]	WEVJ 2025	LiDAR	93.94	86.62	83.54	95.43	91.29	90.03
SPS-RCNN [42]	Sensors 2025	LiDAR	92.79	85.49	83.19	-	-	-
Voxel R-CNN * [9]	AAAI 2021	LiDAR	92.24	84.52	82.54	95.34	91.17	88.68
AVOD [43]	IROS 2018	LiDAR + RGB	84.41	74.44	68.65	86.30	85.44	77.73
MV3D [44]	CVPR 2017	LiDAR + RGB	71.29	62.68	56.56	86.02	76.90	68.49
EPNet [45]	ECCV 2020	LiDAR + RGB	92.28	82.59	80.14	94.22	88.47	83.69
**Ours**	-	LiDAR	**93.27**	**86.64**	84.16	**95.96**	**92.19**	**89.90**

**Table 3:** Comparison with Multimodal Methods on the Validation Set. Best results are highlighted in bold. The bold results indicate the best performance.

Model	Reference	Car.3D (APR40)			Ped.3D (APR40)			Cyc.3D (APR40)		
		Easy	Mod.	Hard	Easy	Mod.	Hard	Easy	Mod.	Hard
LoGoNet [20]	CVPR 2023	92.04	85.04	84.31	70.20	63.72	59.46	91.74	75.35	**72.42**
Bi-Att3DDet [19]	Sensors 2025	95.25	88.32	85.91	74.22	**67.57**	61.14	**95.46**	73.60	68.90
SQD [33]	ACM MM 2024	95.18	84.82	**8** **6.** **07**	-	-	-	-	-	-
SFD [34]	CVPR 2022	**95.47**	88.56	85.74	-	-	-	-	-	-
VirConv-L [35]	CVPR 2023	93.36	**88.71**	85.83	-	-	-	-	-	-
**Ours**	-	93.27	86.64	84.16	**74.57**	67.33	60.78	91.71	**76.37**	71.93

**Table 4:** Ablation Results of Different 3D Backbone Designs for Pedestrian and Cyclist Detection on the KITTI Validation Set. The bold results indicate the best performance.

Model	Ped.3D (APR40)			Cyc.3D (APR40)		
	Easy	Mod.	Hard	Easy	Mod.	Hard
Voxel R-CNN	65.51	59.67	53.73	90.72	70.94	66.88
Voxel R-CNN + SDCM	71.92	64.74	58.18	**91.95**	**74.41**	**69.96**
Voxel R-CNN + SRB	72.27	65.60	59.34	89.13	72.57	68.34
Voxel R-CNN + SDCM + SRB	**72.77**	**65.90**	**60.88**	91.62	73.88	69.49

**Table 5:** Ablation Results of Different Detection Head Designs for Pedestrian and Cyclist Detection on the KITTI Validation Set. The bold results indicate the best performance.

Model	Ped.3D (APR40)			Cyc.3D (APR40)		
	Easy	Mod.	Hard	Easy	Mod.	Hard
Voxel R-CNN	65.51	59.67	53.73	90.72	70.94	66.88
Voxel R-CNN + CSCA	**71.99**	**64.92**	59.18	90.91	74.02	69.56
Voxel R-CNN + CSCA+ FGCA	71.07	64.45	**59.45**	**91.** **38**	**7** **5.15**	**70.57**

**Table 6:** Ablation Results on the KITTI Validation Set Using Different Components. The bold results indicate the best performance.

Method	Module			Car 3D (AP_40_)			Pedestrian 3D (AP_40_)			Cyclist 3D (AP_40_)		
	MSR3 DCNN	Conv Next	CasA _FGCE	Easy	Mod.	Hard	Easy	Mod.	Hard	Easy	Mod.	Hard
Voxel R-CNN				92.24	84.52	82.54	65.51	59.67	53.73	90.72	70.94	66.88
A	√			92.80	86.06	83.64	72.77	65.90	**60.88**	91.62	73.88	69.49
B		√		93.25	86.44	82.02	72.11	66.33	59.68	91.30	74.23	69.65
C			√	93.02	86.19	83.78	71.07	64.45	59.45	91.38	75.15	70.57
D	√		√	93.07	86.43	83.94	72.34	65.64	59.45	91.23	75.50	71.06
E	√	√		92.97	86.24	83.86	73.13	66.24	59.79	**91.74**	74.22	69.80
**Ours**	√	√	√	**93.27**	**86.64**	**84.16**	**74.57**	**67.33**	60.78	91.71	**76.37**	**71.93**
Improvement				+1.03	+2.12	+1.62	+9.06	+7.66	+7.05	+0.99	+5.43	+5.05

**Table 7:** Computational Complexity and Inference Latency Analysis of Different Model Configurations on the KITTI Validation Set √.

MSR3DCNN	ConvNeXt	CasA_FGCE	Car.3D	Ped.3D	Cyc.3D	#Params (M)	Runtime (ms)
√			87.50	66.52	78.33	6.03	47.20
	√		87.24	66.04	78.39	5.76	45.10
		√	87.66	64.99	79.03	11.01	64.30
√	√		87.69	66.39	78.58	6.41	50.90
√		√	87.81	65.81	79.26	11.43	69.60
√	√	√	88.02	67.56	80.00	11.51	70.20

## Data Availability

The original data presented in this study are openly available in KITTI official website: https://www.cvlibs.net/datasets/kitti/ (accessed on 15 August 2025).

## Abbreviations

The following abbreviations are used in this manuscript:

LSCA-RCNN	Large-Kernel Spatial Residual and Cascade Attention in Voxel R-CNN
SRBs	Spatial Residual Blocks
3D	Three-Dimensional
2D	Two-Dimensional
SE	Squeeze-and-Excitation
RoI	Region of Interest
BEV	Bird’s-Eye View
RPN	Region Proposal Network
VFE	Voxel Feature Encoding
BN	Batch Normalization
FGCE	Fine-Grained Group Convolution Enhancement
MSG-CU	Multi-scale Grouped Convolution Unit
MSR3DCNN	Multi-scale Residual 3D CNN
CasA_FGCE	Cascaded Component-Aware RoI Detection Head
SDCM	Spatial–Dimensional Convolutional Modules
FGCA	Fine-Grained Grouped Convolutional Component-Aware
CSCA	Cross-Stage Cross-Attention
